# CBP-JMF: An Improved Joint Matrix Tri-Factorization Method for Characterizing Complex Biological Processes of Diseases

**DOI:** 10.3389/fgene.2021.665416

**Published:** 2021-04-23

**Authors:** Bingbo Wang, Xiujuan Ma, Minghui Xie, Yue Wu, Yajun Wang, Ran Duan, Chenxing Zhang, Liang Yu, Xingli Guo, Lin Gao

**Affiliations:** ^1^School of Computer Science and Technology, Xidian University, Xi'an, China; ^2^School of Humanities and Foreign Languages, Xi'an University of Technology, Xi'an, China

**Keywords:** non-negative matrix factorization, complex biological processes, multi-dimensional genomic data, disease, subtype

## Abstract

Multi-omics molecules regulate complex biological processes (CBPs), which reflect the activities of various molecules in living organisms. Meanwhile, the applications to represent disease subtypes and cell types have created an urgent need for sample grouping and associated CBP-inferring tools. In this paper, we present CBP-JMF, a practical tool primarily for discovering CBPs, which underlie sample groups as disease subtypes in applications. Differently from existing methods, CBP-JMF is based on a joint non-negative matrix tri-factorization framework and is implemented in Python. As a pragmatic application, we apply CBP-JMF to identify CBPs for four subtypes of breast cancer. The result shows significant overlapping between genes extracted from CBPs and known subtype pathways. We verify the effectiveness of our tool in detecting CBPs that interpret subtypes of disease.

## Introduction

Complex biological processes (CBPs) are the coordinated effect of multiple molecules, which result in some functional pathways and the vital processes occurring in living organisms. In addition, the vast amounts of multi-omics data, such as genomics, epigenomics, transcriptomics, proteomics, and metabolomics, can be integrated to understand systems biology accurately (Suravajhala et al., 2016). Hasin et al. (2017) pointed out that a deeper and better understanding of important biological processes and modules can be obtained through multi-omics studies. However, practical tools are still missing to integrate diverse multi-omics data at different biological levels and reveal the CBPs and other problems like the causes of diseases.

Non-negative matrix factorization (NMF) (Lee and Seung, 1999) is a powerful tool for dimension reduction and feature extraction. It has been increasingly applied to diverse fields, including bioinformatics (*e*.*g*., high-dimensional genomic data analysis). For example, Brunet et al. (2004) applied NMF and consensus clustering to the gene expression data of leukemia to discover metagenes and molecular patterns. Xi et al. (2018) detected driver genes from pan-cancer data based on another matrix decomposition framework called matrix tri-factorization. Up to now, several variants of NMF have been proposed, including tri-factorization NMF (Ding et al., 2006), graph-regularized NMF (Cai et al., 2011), joint NMF (Zhang et al., 2012), iNMF (Yang and Michailidis, 2016), *etc*. (more details are in Supplementary Note 1 of the Supplementary Materials). In 2012, jNMF (Zhang et al., 2012) was proposed to identify multi-omics modules by integrating cancer's DNA methylation data, gene expression data, and miRNA expression data. Chen and Zhang (2018) applied joint matrix tri-factorization to discover two-level modular organization from matched genes and miRNA expression data, gene expression data, and drug response data.

Omics data across the same samples contain signal values from expression counts, methylation levels, and protein concentrations, which control biological systems, resulting in so-called multi-dimensional genomic (MG) data. The natural representation of these diverse MG data is a series of matrices with measured values in rows and individual samples in columns. Recently, there are integrative analysis tools based on NMF technique that reveal low-dimensional structure patterns. The low-dimensional structure patterns reflect CBPs and sample groups while preserving as much information as possible from high-dimensional MG data (Stein-O'Brien et al., 2018).

In general, most particular matrix factorization techniques are being developed to enhance their applicability to specific biological problems. Meanwhile, the applications to represent disease subtypes (Biton et al., 2014) and cell types (Fan et al., 2016) have created an urgent need for sample grouping and associated CBP-inferring tools. Moreover, cancer and other complex diseases are heterogeneous, *i*.*e*., there are various subgroups for a cancer or a complex disease. The study of the heterogeneity of cancer and complex diseases will help us understand the disease further and provide better opportunities to disease treatment (Xi et al., 2020). To address this issue, we extend traditional jNMF and develop CBP-JMF, an improved joint matrix tri-factorization framework for characterizing CBPs that represent sample groups, and implement a Python package. This package takes labeled samples as the prior information and integrates MG data (*e*.*g*., copy number variation, gene expression, microRNA expression, and/or molecule interaction network) to identify the underlying CBPs which characterize the specific functional properties of each group. CBP-JMF can be used to mark unlabeled samples with groups of known labels. For ease of use, CBP-JMF can recommend reasonable parameter settings for users. CBPs found by CBP-JMF are connected network markers, and they are distinguished between sample groups. These markers usually have specific biological functions and play important roles in phenotypes. As an example, CBPs for subtypes of breast cancer are obtained by CBP-JMF, but they may not have been collected in any reference database yet.

The rest of this paper is organized as follows. Section “Framework of CBP-JMF” deals with the problem formulation of CBP-JMF and the implementation of it. Then, Section “Results” exemplifies our approach by applying CBP-JMF to identify CBPs for different subtypes of breast cancers and compares the results of classifying unlabeled samples with CBP-JMF and its several variants. Finally, Section “Discussion” discusses our results and lists our expectations of our method and the limitations of it. Section “Conclusions” highlights our method.

## Framework of CBP-JMF

### Problem Definition

Given a non-negative matrix **X** ∈ **R**^*m*×*n*^, it can be factorized into three non-negative matrix factors based on matrix tri-factorization: **X** ≈ **USV**, where **U** ∈ **R**^*m*×*k*^, **S** ∈ **R**^*k*×*k*^, and **V** ∈ **R**^*k*×*n*^. Factored matrix **S** cannot only absorb scale difference between **U** and **V** but also indicates relationships between the identified *k* modules.

**Figure 1:**
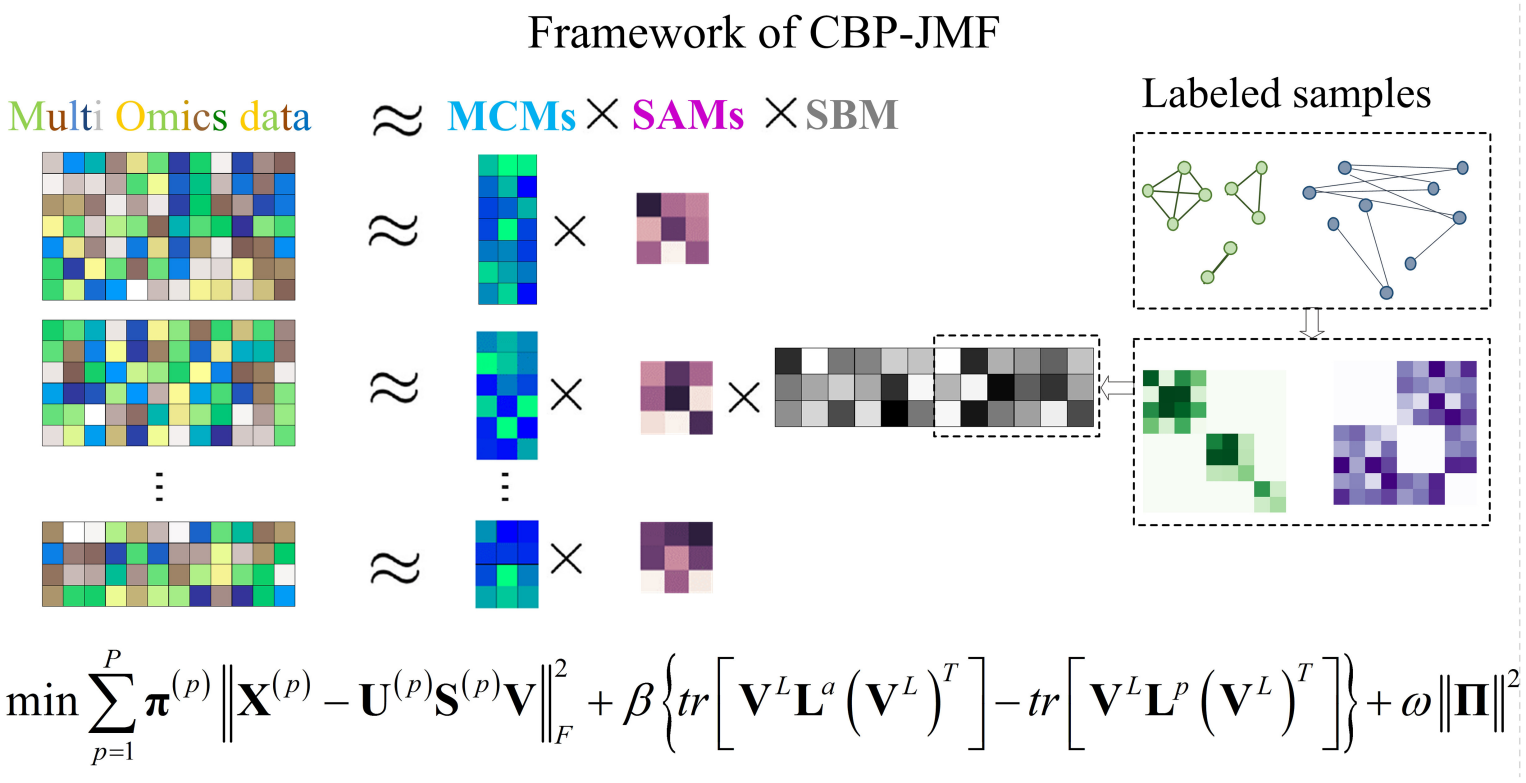
Illustration of the framework and optimization objective function of complex biological processes–joint matrix tri-factorization.

In CBP-JMF, given a MG dataset composed of *P* omics, it can be presented by multiple matrices **X**^(1)^, **X**^(2)^, ..., **X**^(*P*)^, as illustrated in Figure 1. For each matrix, the rows indicate molecules like genes, and the columns indicate samples; the values in it are related to the meaning of omics. If **X**^(*p*)^ (*p* ∈ [1, *P*]) is a matrix of gene expression data, ${\text{X}}_{ij}^{\left(p\right)}$ represents the expression value of the gene in the *i*-th row on the *j-*th sample. Basically, each non-negative matrix **X**^(*p*)^ ∈ **R**^*m*×*n*^, *p* = 1, 2, ..., *P* is factorized into three non-negative matrix factors based on matrix tri-factorization: **X**^(*p*)^ ≈ **U**^(*p*)^**S**^(*p*)^**V**, where molecular coefficient matrix (MCM) **U**^(*p*)^ ∈ **R**^*m*×*k*^ and sample basis matrix (SBM) **V** ∈ **R**^*k*×*n*^ are the pattern indicator matrices of *k* CBPs and *k* sample groups, respectively. Scale absorbing matrix (SAM) **S**^(*p*)^ ∈ **R**^*k*×*k*^ explores the relationships between them. Furthermore, MCM describes the structure pattern between molecules (*e*.*g*., genes), SBM indicates the structure pattern between samples, and SAM absorbs the difference of scales between MCM and SBM (Figure 1). Each column of the MCM infers a latent feature associated with a CBP, and the continuous values in it represent the relative contribution of each molecule in the CBP. Meanwhile, each row of the SBM describes the relative contributions of the samples to a latent feature. The sample groups can be detected by comparing the relative weights in each row of the SBM.

Overall, **X**^(1)^, **X**^(2)^, ..., **X**^(*P*)^ can be jointly factorized into specific **U**^(1)^, **U**^(2)^, ..., **U**^(*P*)^, **S**^(1)^, **S**^(2)^, ..., **S**^(*P*)^, and a common matrix **V**. **X**^(1)^, **X**^(2)^, ..., **X**^(*P*)^ are across the same samples, and **V** reveals consistent sample groups of multi-omics data. In CBP-JMF, **V** can be divided into **V**^*L*^ and **V**^*UL*^ according to input data, where L and UL mean “labeled” samples and “unlabeled” samples, respectively.

### Objective Function of CBP-JMF

Considering that different datasets may play different roles in data integration, we adopted a method that can learn the weights of different input data through a weighted joint tri-NMF:

(1) $$\begin{array}{l} \min\sum_{p=1}^{P}\pi^{\left(p\right)}{\left\|{\text{X}}^{\left(p\right)}-{\text{U}}^{\left(p\right)}{\text{S}}^{\left(p\right)}\text{V}\right\|}_{F}^{2}+\omega {\left\|\Pi \right\|}^{2}\\ s.t.\;\pi^{\left(p\right)}>0,\sum_{p=1}^{P}\pi^{\left(p\right)}=1 \end{array}$$

where Π = (π^(1)^, π^(2)^, ..., π^(*P*)^). CBP-JMF differentiates the importance of datasets by the weight constraint ||Π||^2^, and π^(*p*)^ will get a weight to represent the contribution of data **X**^(*p*)^ to objective function after optimization. If **X**^(*p*)^ contributes to the optimization of cost function, then it will be given a higher weight π^(*p*)^, or if **X**^(*p*)^ contains lots of noises which hinder the optimization of objective function, it will be given a lower weight π^(*p*)^.

In addition, **V** can be divided into labeled **V**^*L*^ and unlabeled **V**^*UL*^ parts according to the labeled samples and unlabeled samples. In order to learn the correlation between labeled samples, we use a graph Laplacian to represent the distance of labeled sample in latent space (Guan et al., 2015). We use Equations (2) and (3) to denote the distance between labeled samples from the same class and different class in the learned latent space, respectively,

(2) $$\begin{array}{r} \overset{N^{L}}{\underset{i=1}{\sum }}\overset{N^{L}}{\underset{j=1}{\sum }}{\text{W}}_{ij}^{a}\|{\text{v}}_{i}^{L}-{\text{v}}_{j}^{L}{\|}_{2}^{2}=tr\left[{\text{V}}^{L}{\text{L}}^{a}{\left({\text{V}}^{L}\right)}^{T}\right] \end{array}$$

(3) $$\begin{array}{r} \overset{N^{L}}{\underset{i=1}{\sum }}\overset{N^{L}}{\underset{j=1}{\sum }}{\text{W}}_{ij}^{p}\|{\text{v}}_{i}^{L}-{\text{v}}_{j}^{L}{\|}_{2}^{2}=tr\left[{\text{V}}^{L}{\text{L}}^{p}{\left({\text{V}}^{L}\right)}^{T}\right]\; \end{array}$$

where *N*^*L*^ is the number of labeled samples in **V**, and **W**^*a*^ (**W**^*affinity*^) and **W**^*p*^ (**W**^*penalty*^) are the weighted adjacency matrices (see Supplementary Note 2 in SM) corresponding to intra-group and inter-group samples respectively. **L**^*a*^ (**L**^*affinity*^) and **L**^*p*^ (**L**^*penalty*^) are the Laplacian matrix of **W**^*a*^ and **W**^*p*^, respectively, where **L**^*a*^=**D**^*a*^ − **W**^*a*^, **L**^*p*^=**D**^*p*^ − **W**^*p*^, **D**^*a*^=$\sum_{j=1}^{N^{L}}{\text{W}}_{ij}^{a}$. In machine learning, people try to make samples from the same class near each other in the learned latent space and samples from different class far from each other. This principle can be written as

(4) $$\begin{array}{r} \min\left(tr\left[{\text{V}}^{L}{\text{L}}^{a}{\left({\text{V}}^{L}\right)}^{T}\right]-tr\left[{\text{V}}^{L}{\text{L}}^{p}{\left({\text{V}}^{L}\right)}^{T}\right]\right) \end{array}$$

Combining weighted joint tri-NMF and the constraints of correlation between labeled samples mentioned above, we give the formulation of the optimization objective function of CBP-JMF as follows (Figure 1):

(5) $$\begin{array}{l} \underset{{\left\{U^{\left(p\right)}\right\}}_{p=1}^{P},{\left\{S^{\left(p\right)}\right\}}_{p=1}^{P},V}{\min}\sum_{p=1}^{P}\pi^{\left(p\right)}{\left\|X^{\left(p\right)}-U^{\left(p\right)}S^{\left(p\right)}V\right\|}_{F}^{2}\\ +\beta \left\{tr\left[V^{L}L^{a}{\left(V^{L}\right)}^{T}\right]-tr\left[V^{L}L^{p}{\left(V^{L}\right)}^{T}\right]\right\}+\omega {\left\|\Pi \right\|}^{2}\\ s.t.\forall p,U_{ij}^{\left(p\right)}\geq 0,V_{ij}\geq 0,\pi^{\left(p\right)}\geq 0,\sum_{p=1}^{P}\pi^{\left(p\right)}=1 \end{array}$$

Parameters β and ω represent the importance of the graph Laplacian regularization and weight constraint ||Π||^2^. In total, each **X**^(*p*)^ is factorized into individual molecular matrix **U**^(*p*)^ and scale matrix **S**^(*p*)^ and a common sample matrix **V**. We allowed all matrices to share the same sample matrix **V** for finding common factors in MG data. There is only a part of samples labeled (subtype or subpopulation or subgroup is known as prior information); we incorporate this prior information with graph Laplacian. We can also learn the weights of different input data to conclude the roles that different data matrices play in CBP-JMF.

### Optimization and Update Rules of CBP-JMF

To solve the problem of factorization **X** ≈ **USV**, we firstly randomly initialize the solution of **U**, **S**, and **V** and then apply iterative multiplicative updates as the optimization approach similar to EM algorithms (Dempster et al., 1977). The optimization procedure of CBP-JMF is as follows.

**Algorithm 1:** The CBP-JMF algorithm.

**Input:**
*P* data matrices *X*^(1)^, *X*^(2)^, ..., *X*^(*P*)^, parameters *β* *ω*
**Output:**
*P* basis matrices **U**^(1)^, **U**^(2)^, ..., **U**^(*P*)^, *P* relation matrices **S**^(1)^, **S**^(2)^, ..., **S**^(*P*)^, factor matrices **V**, weight vector Π = (*π*^(1)^, *π*^(2)^, ..., *π*^(*P*)^)
1: **Begin**
2: Initialize**U**^(1)^, **U**^(2)^, ..., **U**^(*P*)^, **S**^(1)^, **S**^(2)^, ..., **S**^(*P*)^, V
3: Initialize $\left(\pi^{\left(1\right)},\pi^{\left(2\right)},...,\pi^{\left(P\right)}\right)\text{=}\left(\frac{1}{P},\frac{1}{P},...,\frac{1}{P}\right)$
4: **loop**
5: **for** *p*=1 to *P* **do**
6: Fix V, update ****U**^(*p*)^**, ****S**^(*p*)^**
7: **end for**
8: Fix **U**^(1)^, **U**^(2)^, ..., **U**^(*P*)^, update **V**^*L*^
9: Fix **U**^(1)^, **U**^(2)^, ..., **U**^(*P*)^, update **V**^*UL*^
10: **for** *p*=1 to *P* **do**
11: Fix **U, S, V**, compute $c^{\left(p\right)}=\|{\text{X}}^{\left(p\right)}-{\text{U}}^{\left(p\right)}{\text{S}}^{\left(p\right)}\text{V}{\|}_{F}^{2}$
12: **end for**
13: **Update** Π
14: **break** loop if convergence
15: **End**

To clarify the update rules of the objective function of CBP-JMF, we define $O\left(\text{U},\text{V},\text{S},\text{Π}\right)=\overset{P}{\underset{p=1}{\sum }}\pi^{\left(p\right)}\|{\text{X}}^{\left(p\right)}-{\text{U}}^{\left(p\right)}{\text{S}}^{\left(p\right)}\text{V}{\|}_{F}^{2}+\beta \left\{tr\left[{\text{V}}^{L}{\text{L}}^{a}{\left({\text{V}}^{L}\right)}^{T}\right]-tr\left[{\text{V}}^{L}{\text{L}}^{p}{\left({\text{V}}^{L}\right)}^{T}\right]\right\}+\omega {\left\|\text{Π}\right\|}^{2}$. Firstly, we fix **V** and **S** and update **U**; then, we can get the Lagrange function and let Ψ be the Lagrange multiplier for the constraints ${\text{U}}_{ij}^{\left(p\right)}>0$.

(6) $$\begin{array}{r} L\left({\text{U}}^{\left(P\right)}\right)=O\left({\text{U}}^{\left(P\right)}\right)+tr\left({\text{Ψ}}^{T}{\text{U}}^{\left(P\right)}\right) \end{array}$$

The partial derivatives of *L*(**U**^(*P*)^) with **U** is:

(7) $$\begin{array}{r} \frac{\partial L\left({\text{U}}^{\left(P\right)}\right)}{\partial {\text{U}}^{\left(P\right)}}=\text{−}2{\text{X}}^{\left(p\right)}{\text{V}}^{T}{\left({\text{S}}^{\left(p\right)}\right)}^{T}+2{\text{U}}^{\left(P\right)}{\text{S}}^{\left(p\right)}\text{V}{\text{V}}^{T}{\left({\text{S}}^{\left(p\right)}\right)}^{T}+\text{Ψ} \end{array}$$

Based on the KKT conditions Ψ_*ij*_**U**_*ij*_ = 0, we can get the following update rules:

(8) $$\begin{array}{r} {\text{U}}^{\left(P\right)}\leftarrow {\text{U}}^{\left(P\right)}\circ \frac{{\text{X}}^{\left(P\right)}{\text{V}}^{\text{T}}{\left({\text{S}}^{\left(p\right)}\right)}^{T}}{{\text{U}}^{\left(P\right)}{\text{S}}^{\left(p\right)}\text{V}{\text{V}}^{\text{T}}{\left({\text{S}}^{\left(p\right)}\right)}^{\text{T}}} \end{array}$$

Similarly, we can get the update rules for **W**, **V**^*L*^, and **V**^*UL*^:

(9) $$\begin{array}{r} {\text{S}}^{\left(P\right)}\leftarrow {\text{S}}^{\left(P\right)}\circ \frac{{\left({\text{U}}^{\left(p\right)}\right)}^{T}{\text{X}}^{\left(p\right)}{\text{V}}^{T}}{{\left({\text{U}}^{\left(p\right)}\right)}^{T}{\text{U}}^{\left(P\right)}{\text{S}}^{\left(p\right)}\text{V}{\text{V}}^{T}} \end{array}$$

(10) $$\begin{array}{r} {\text{V}}^{L}\leftarrow {\text{V}}^{L}\circ \frac{\overset{P}{\underset{p=1}{\sum }}\pi^{\left(p\right)}\left({\left({\text{S}}^{\left(p\right)}\right)}^{T}{\left({\text{U}}^{\left(p\right)}\right)}^{T}{{\text{X}}^{L}}^{\left(p\right)}\right)+\beta {\text{V}}^{L}\left({\text{D}}^{p}+{\text{S}}^{a}\right)}{\overset{P}{\underset{p=1}{\sum }}\pi^{\left(p\right)}{\left({\text{S}}^{\left(p\right)}\right)}^{T}{\left({\text{U}}^{\left(p\right)}\right)}^{T}{\text{U}}^{\left(p\right)}{\text{S}}^{\left(p\right)}{\text{V}}^{L}+\beta {\text{V}}^{L}\left({\text{D}}^{a}+{\text{S}}^{p}\right)} \end{array}$$

(11) $$\begin{array}{r} {\text{V}}^{UL}\leftarrow {\text{V}}^{UL}\circ \frac{\overset{P}{\underset{p=1}{\sum }}\pi^{\left(p\right)}\left({\left({\text{S}}^{\left(p\right)}\right)}^{T}{\left({\text{U}}^{\left(p\right)}\right)}^{T}{{\text{X}}^{UL}}^{\left(p\right)}\right)}{\overset{P}{\underset{p=1}{\sum }}\pi^{\left(p\right)}{\left({\text{S}}^{\left(p\right)}\right)}^{T}{\left({\text{U}}^{\left(p\right)}\right)}^{T}{\text{U}}^{\left(p\right)}{\text{S}}^{\left(p\right)}{\text{V}}^{UL}} \end{array}$$

As for updating of π, when **U**,**V**, and **S** are fixed, minimization of *O*(π) is a convex optimization, and we use convex optimization toolbox to update π.

### CBPs Obtained From CBP-JMF

Values in each column of **U**^(*p*)^ represent the relative contribution of each molecule in each module, and values in each row of **V** represent the degree of each sample involved in each module. According to the rules of matrix multiplication, the *i*-th column of basis matrix **U**^(*p*)^, *p* = 1, 2, ..., *P* corresponds to the *i*-th row of coefficient matrix **V**, so there is a one-to-one correspondence between subtype and multi-omics module discovered from the columns of **U**^(*p*)^ matrix. Firstly, we need to know the relationship between *k* modules and subtypes by counting each subtype's value in each module from **V**^(*p*)^ matrix (see Supplementary Note 3 in Supplementary Material).

To select features associated with each module, CBP-JMF calculates the z-scores of each molecule for each column vector of **U**^(*p*)^ as $z=\left(x-\bar{x}\right)/S_{x}$, where $\bar{x}=\frac{1}{n}\sum_{i}x_{i},\;S_{x}^{2}=\frac{1}{n-1}\sum_{i}{\left(x_{i}-\bar{x}\right)}^{2}$. Let ${\text{u}}_{j}^{\left(p\right)}$ be the *j-*th column of **U**^(*p*)^ and infer a latent feature associated with *j-*th CBP. The continuous value ${\text{u}}_{ij}^{\left(p\right)}$ represents the relative contribution of molecule *i* in the *j*-th CBP. ${\text{u}}_{ij}^{\left(p\right)}$ can be regarded as *x*_*i*_, and the length of ${\text{u}}_{j}^{\left(p\right)}$ can be regarded as *n* in Equation (12). CBP-JMF calculates a z-score for each value in ${\text{u}}_{j}^{\left(p\right)}$ and obtains CBP's members through a given cutoff (*z*-score >2 in our tests). Then, they are mapped to a built-in molecule interaction network (see “Section ‘Results”') to extract their connected components as the final CBP.

## Results

We applied CBP-JMF to BRCA with multi-omics data. The reason we chose BRCA as example is that breast cancer is a heterogeneous complex disease, and it is the most commonly occurring cancer. BRCA is also a type of cancer that can be divided into smaller groups based on certain characteristics of the cancer cells. Distinct complex biological processes represent different subtypes. Characterizing the processes can provide us comprehensive insights into the mechanisms of how multiple levels of molecules interact with each other and the heterogeneity of breast cancers.

### Data

Firstly, we downloaded the Gene Expression (GE) data, miRNA expression (ME) data, and copy number variation (CNV) data across the same set of 738 breast cancer samples from UCSC Xena (Goldman et al., 2018). Secondly, we obtained the sample label information which is classified by PAM50 from The Cancer Genome Atlas Network (Koboldt et al., 2012). Among 738 samples, there are 522 breast cancer samples with labels, including 231 luminal A, 127 luminal B, 98 triple negative/basal-like, 58 HER2-enriched, and eight normal-like. Thirdly, we filtered out some samples, in which more than 90% of the genes have an expression value of zero. For genes and miRNAs, we filtered the genes and miRNAs with an expression value of zero in more than 20% of the samples. Fourthly, we did differential expression analysis for genes using edgeR package (Robinson et al., 2009) in R with *P*-value < 0.01 and |log(fold change)|> 0.5 to filter out genes which are not associated with breast cancer. Fifthly, we imputed missing miRNA data using knnimpute package in MATLAB. About the CNV data, the GISTIC2 (Mermel et al., 2011) thresholded the estimated values of CNV to −2, −1, 0, 1, and 2, which represent homozygous deletion, single copy deletion, diploid normal copy, low-level copy number amplification, or high-level number amplification. Finally, we obtained the GE data **X**^(1)^ ∈ **R**^2913×725^ and ME data **X**^(2)^ ∈ **R**^516×725^. Among 725 samples, 179 samples are marked with subtype labels (80 luminal A, 38 luminal B, 39 basal-like, 22 HER2-enriched) and shared between GE, ME, and CNV datasets. Furthermore, we calculated the Pearson correlation of 179 labeled samples using CNV data to construct **W**^*a*^ ∈ **R**^179×179^, **W**^*p*^ ∈ **R**^179×179^, and their Laplacian matrices to form the graph Laplacian regularization $tr\left[{\text{V}}^{L}{\text{L}}^{a}{\left({\text{V}}^{L}\right)}^{T}\right]-tr\left[{\text{V}}^{L}{\text{L}}^{p}{\left({\text{V}}^{L}\right)}^{T}\right]$.

### Complex Biological Processes for Breast Cancer Subtypes

In our example, we set parameters *k* = 4, β = 10, and ω = 100, 000. Other parameters and more details can be found in Supplementary Note 2 of Supplementary Material. As a result, we obtained unique matrices **U**^(1)^ ∈ **R**^2913×4^, **U**^(2)^ ∈ **R**^516×4^, **S**^(1)^ ∈ **R**^4×4^, and **S**^(2)^ ∈ **R**^4×4^ and a common matrix **V** ∈ **R**^4 ×725^.

**Figure 2:**
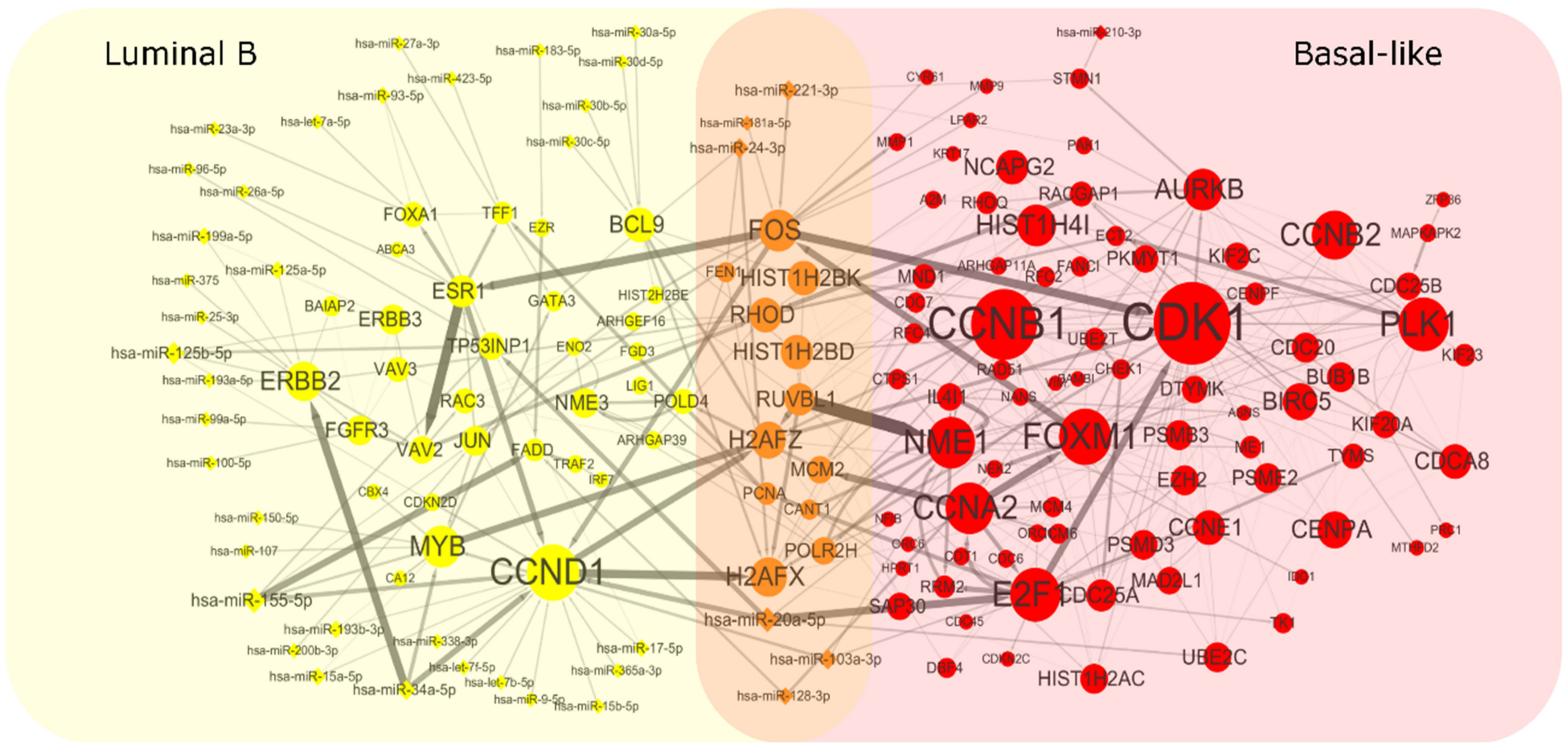
Complex biological processes of luminal B and basal-like subtype. We mapped the genes and miRNAs obtained from luminal B's module and basal-like's module to an integrated gene regulation network. The network was obtained through integrating three databases including Reactom, Kyoto Encyclopedia of Genes and Genomes, and Nci-PID Pathway Interaction Database. The interactions between genes and miRNAs were obtained from miRTarBase. The size of the node is proportional to the size of the degree. The thickness of the edges indicates the strength of the regulatory relationship expressed by the Pearson correlation coefficient between microRNA and gene.

To get heterogeneous CBPs (Supplementary Table 1), directed regulatory pathways containing miRNAs and genes, which correspond to each cancer subtype we put subtype-specific multi-omics modules obtained from matrix **U**^(*p*)^, *p* = 1, 2 onto an integrated gene regulation network from Reactome (Croft et al., 2014), Kyoto Encyclopedia of Genes and Genomes (KEGG) (Kanehisa and Goto, 2000), and Nci-PID pathway (Schaefer et al., 2009). Then, we add directed regulatory edges from miRNA to the gene supported by miRTarBase (Chou et al., 2018). Finally, we extracted the maximum connected component of the regulation network and showed the discovered characteristic CBPs underlying luminal B and basal-like subtypes in Figure 2.

**Figure 3:**
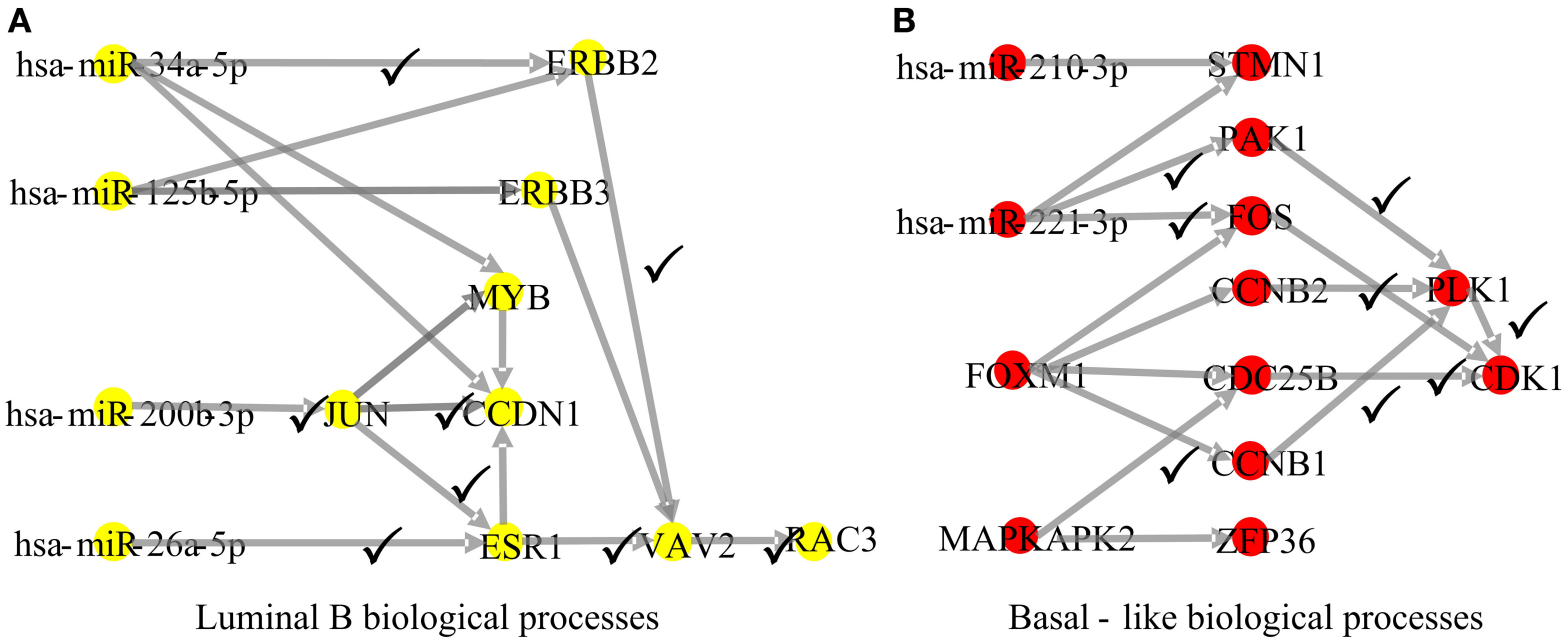
Part of complex biological processes luminal B and basal-like. The edges with checkmarks are the interactions that have been documented. **(A)** Luminal B's biological processes: luminal subtypes are driven by the estrogen/ER pathway. Among all nodes, ERBB2, ERBB3, and ESR1 are involved in the estrogen/ER pathway. **(B)** Basal-like's biological processes: basal-like subtype is driven by the deregulation of various signaling pathways (Notch, MAPK, FoxO signaling pathway, and Wnt/beta-catenin). Among all nodes, MAPKAPK2, CDC25B, CCNB1, CCNB2, PAK1, and STMN1 are known to exist in multiple signaling pathways.

To explore whether the genes in the CBPs of luminal B and basal-like subtype have significant biological importance or not, we performed an enrichment analysis with all 124 genes from Figure 2 across six datasets. The datasets are from OMIM (Hamosh et al., 2005), CGC (Futreal et al., 2004), virhostome, kinome (Manning et al., 2002), drug target (Wishart et al., 2008), KEGG pathway of BRCA (Kanehisa and Goto, 2000). Genes associated with breast cancer or breast tissue in the six datasets are selected as the set of enrichment analysis. Genes extracted through CBP-JMF have significant overlapping with known datasets (Table 1). Furthermore, for each subtype's CBP, functional enrichment analysis (Supplementary Figure 4) shows that four CBPs are mainly enriched in known biological processes and pathways associated with breast cancer, such as cell cycle and various signaling pathways (including p53 signaling pathway and estrogen pathway). However, each CBP also has its specific biological processes and path. This may explain differences between subtypes. As a demonstration, we take the CBPs of luminal B and basal-like as example. Based on the study of the subtypes of BRCA, luminal B is mainly driven by the estrogen/ER pathway (Zhang et al., 2014). In our discovered CBPs, we found several CBPs containing genes like ERBB2, ERBB3, and ESR1 that are related to the estrogen/ER pathway. Besides that, through literature review, miRNAs in luminal B's CBP can regulate the estrogen/ER pathway, such as miR-34a, miR-125b, miR-200b, and so on (Figure 3, Table 2). In addition, basal-like subtype is mainly driven by the deregulation of various signaling pathways including Notch, MAPK, and wnt/β-catenin signaling pathway (King et al., 2012). In our discovered CBPs, we found genes involved in the above-mentioned pathways, such as MAPKAPK2, CDC25B, PLK1, and so on. Besides that, we also found that miRNAs in CBPs of basal-like, such as miR-221 and miR-210, may regulate the genes above in basal-like subtype (Figure 3, Table 3). In summary, subtype-specific biological processes can be identified by CBP-JMF, and CBP-JMF can help users discover potential biological targets.

**Table 1:** Enrichment analysis of the extracted module gene across six datasets.

**Dataset**	**Online mendelian inheritance in man**	**CGC**	**Virhostome**	**Kinome**	**Drug target**	**BRCA pathway**
Total	51	43	947	516	61	102
Overlapped nodes	2	5	13	6	3	6
*P*-value	0.049	0.0003	0.007	0.008	0.010	0.012

**Table 2:** Evidences of luminal B's complex biological processes.

**Interactions**	**Literatures**	**Descriptions**
miR-34a->ERBB2	Wang et al., 2017	MiR-34a modulates ErbB2 in breast cancer
ERBB2->VAV2	Wang et al., 2006	ErbB2 colocalizes with Vav2 *via* activation of PI3K
VAV2->RAC3	Rosenberg et al., 2017	Vav2 promotes Rac3 activation at invadopodia
miR-200b->JUN	Jin et al., 2017	MiR-200b upregulates JUN in breast cancer
JUN->CCND1	Cicatiello et al., 2004	CCND1 promoter activation by estrogens in human breast cancer cells is mediated by the recruitment of a c-Jun/c-Fos/estrogen receptor
JUN->ESR1	Stossi et al., 2012	The activation of ESR1 gene locus in a process that was dependent upon activation and recruitment of the c-Jun transcription factor
miR-26a->ESR1	Howard and Yang, 2018	MiR-26a modulates ESR1 in breast cancer
ESR1->VAV2	Grassilli et al., 2014	ESR1 upregulates VAV2 in breast cancer cell lines

**Table 3:** Evidences of basal-like's complex biological processes.

**Interactions**	**Literatures**	**Descriptions**
CCNB1(CCNB2)->PLK1->CDK1	Li et al., 2019	CCNB1 (CCNB2), PLK1, and CDK1 have interactions in chicken breast muscle
miR221->FOS	Yao et al., 2016	miR221 modulates FOS
miR221->PAK1	Ergun et al., 2015	miR221 modulates PAK1 in breast cancer cell lines
PAK1->PLK1	Maroto et al., 2008	PAK1 regulates PLK1
MAPKAPK2->CDC25B	MAPK signaling pathway	MAPKAPK2 and CDC25B are involved in MAPK signaling pathway
CDC25B->CDK1	Timofeev et al., 2010	Timely assembly of CDK1 required CDC25B

**Figure 4:**
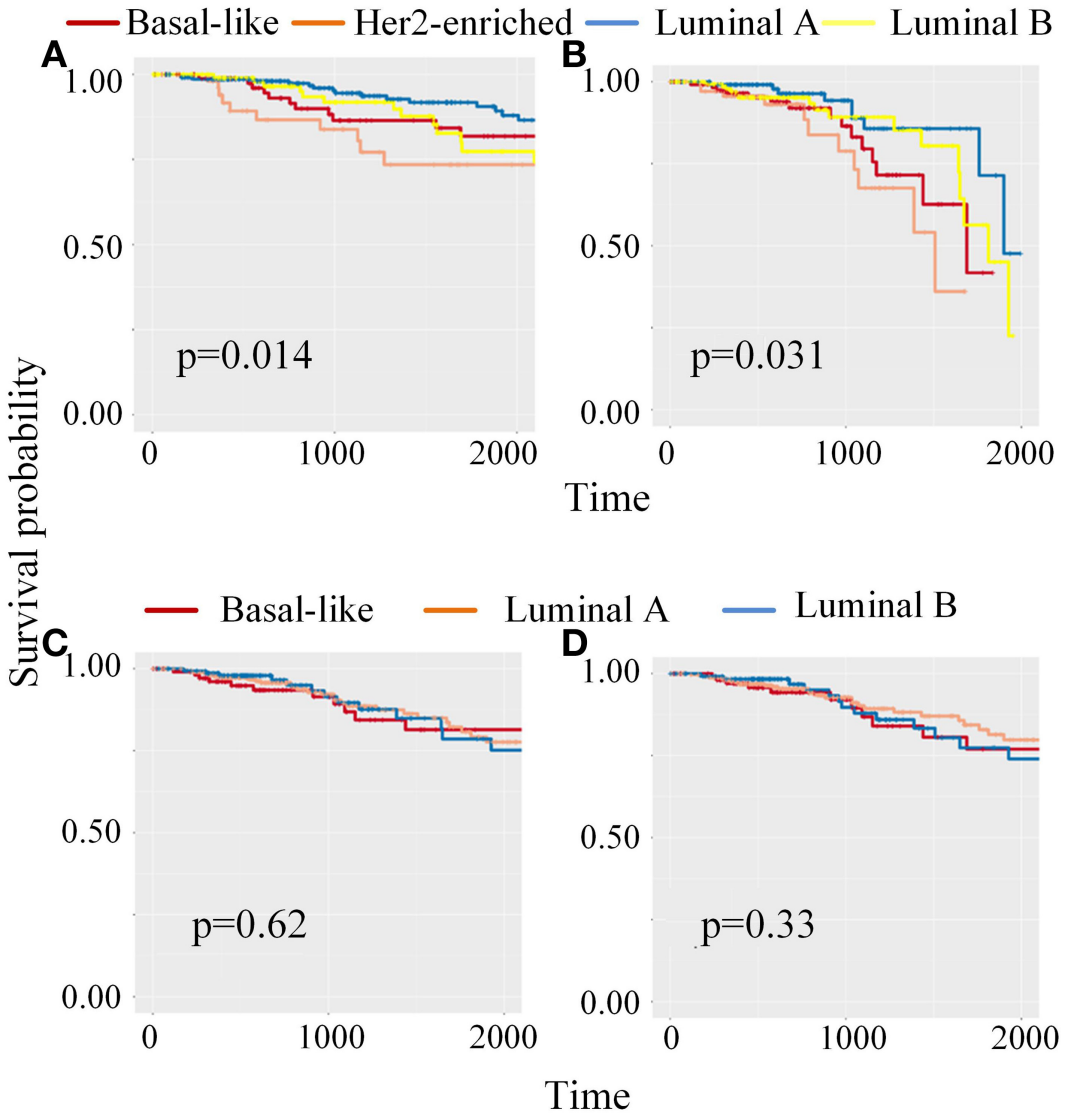
Kaplan–Meier (K–M) survival analysis for patients which are classified using different methods. **(A)** KM survival curve for labeled samples, whose subtypes are known in advance. **(B)** KM survival analysis for unlabeled samples, which are classified using complex biological processes–joint matrix tri-factorization (CBP-JMF) on mRNA expression and miRNA expression data. **(C)** KM survival analysis for unlabeled patients, which are classified using CBP-JMF only on mRNA expression. **(D)** KM survival analysis for unlabeled patients, which are classified on mRNA expression and miRNA expression data without graph embedding regularization.

Meanwhile, to classify unlabeled samples into subtypes, CBP-JMF returned predicted labels for unlabeled samples (Supplementary Note 4 in Supplementary Material). Figure 4 shows the Kaplan–Meier (KM) survival analysis using survival package (Therneau, 2015) on unlabeled samples based on their clinical data in TCGA. We compared our results with other NMF methods (Supplementary Note 4 of Supplementary Material) and found that CBP-JMF achieves more accurate subtype classification results. Unlabeled samples are classified by using GE data and ME data. Figure 4 indicates that the survival analysis for unlabeled samples has the most significant Cox (Lin and Zelterman, 2002) *p*-value 0.031 and similar survival curves like the labeled samples. This proves that the CBP-JMF framework is useful for cancer subtyping, as the framework incorporates integration of multi-omics data and samples' prior information.

## Discussion

Understanding CBPs is vital to help us further understand the development of disease and intervene in the disease. NMF is an effective tool for dimension reduction and data mining in high-throughput genomic data. In this paper, we proposed CBP-JMF, an improved method of multi-view data analysis. It is designed for heterogeneous biological data based on NMF. Moreover, we created an easy-to-use package in Python. CBP-JMF analyzes multi-dimensional genomic data across the same samples integrally. Our method can discover CBPs that underlie sample groups and classify unlabeled samples through learning the relationship between labeled samples.

We tested this framework on the gene expression data and miRNA expression data of BRCA. CBP-JMF discovered subtype-specific biological processes and classified unlabeled samples into four subtypes. We did survival analysis and function analysis, and the results showed that CBP-JMF has great performance. Furthermore, CBP-JMF is a weighted joint tri-NMF framework in essence. We expect that it can be applied to vast fields including disease subtypes, cell types, and population stratification. Meanwhile, we expect that CBP-JMF can be used to identify hub genes or predict the association between genes or non-coding mRNA and diseases by integrating a variety of data. Though CBP-JMF is efficient to uncover CBPs by integrating multi-omics data, CBP-JMF must integrate different multi-omics data that have the same samples. This weakness limits the use of more types of data and integrates more information to obtain more significant results.

## Conclusions

In this article, we develop CBP-JMF, a matrix tri-factorization and weighted joint integration tool, for detecting CBPs, which characterize prior disease subtypes and cell groups in Python. We improve its usability by estimating the parameters, such as determining the number of features through consensus clustering. CBP-JMF always gives reference values of all parameters. In applications, CBP-JMF characterizes the CBPs of four subtypes of BRCA based on gene and miRNA expression data from TCGA, and we find the significantly different functional pathways that characterized luminal B and basal-like subtypes.

## Data Availability Statement

The datasets presented in this study are publicly available and the addresses for finding them are listed within the article. Prediction results and a reference implementation of CBP-JMF in Python are available at: https://github.com/wangbingbo2019/CBP-JMF.

## Author Contributions

BW, YWu, and XM conceived and designed the experiments. YWu and MX performed the experiments. XM, RD, CZ, LY, XG, and LG analyzed the data. BW, YWu, XM, and YWa proofread the paper. All authors contributed to the article and approved the submitted version.

## Conflict of Interest

The authors declare that the research was conducted in the absence of any commercial or financial relationships that could be construed as a potential conflict of interest.
