# Supplementary material for: CBP-JMF: An Improved Joint Matrix Tri-Factorization Method for Characterizing Complex Biological Processes of Diseases
Source: Front Genet. 2021 Apr 23;12:665416. doi: 10.3389/fgene.2021.665416 (PMC8103031; doi:10.3389/fgene.2021.665416)
Supplement: Supplementary file 1 [file Data_Sheet_1.docx]

Supplementary Material

# Supplementary Notes

## Supplementary Note 1. Brief Review of NMF

Given a set of n-dimensional data vectors, all of which are placed in the columns of an m×n matrix $\mathbf{X}$. $\mathbf{X}$ can be decomposed into an m×k matrix $\mathbf{U}$ and a k×n matrix $\mathbf{V}$ based on two-factor NMF, k is chosen to be smaller than n or m, so that $\mathbf{U}$ and $\mathbf{V}$ are smaller than the original matrix $\mathbf{X}$. It can be rewritten as $\mathbf{X}\approx\mathbf{UV}$, where $\mathbf{x}$ and $\mathbf{v}$ are columns of $\mathbf{X}$ and $\mathbf{V}$. In this way, each column vector $\mathbf{x}$ is approximated by a linear combination of the columns of $\mathbf{U}$, weighted by the components of $\mathbf{v}$, so original matrix $\mathbf{X}$ is explained by a positive linear combination of basis vectors (the columns of $\mathbf{U}$). And a good approximation can discover latent structure in the original matrix. To measure the distance between $\mathbf{X}$ and $\mathbf{UV}$, two types of cost functions are often used. One is based on the Euclidean distance $\text{‖}\mathbf{A}-\mathbf{B}\text{‖}^{2}=\sum_{ij} \left(\mathbf{A}_{ij}-\mathbf{B}_{ij}\right)^{2}$, another useful measure is based on the Kullback-Leibler divergence $D(\mathbf{A}\text{‖}\mathbf{B})=\sum_{ij} \left(\mathbf{A}_{ij}\log\frac{\mathbf{A}_{ij}}{\mathbf{B}_{ij}}-\mathbf{A}_{ij}+\mathbf{B}_{ij}\right)$, Lee et al. (Lee and Seung, 1999) proved that cost functions are convex and provide multiplicative update rules which converge to a local minimum. Moreover, multiplicative update rules have still been widely used. Besides, to accelerate the convergence rate, several new update rules have been proposed (Wang and Zhang, 2013). One popular method is gradient descent algorithms with additive update rules. Other techniques**,** such as conjugate gradient, projected gradient, are also in consideration. In actual application, matrix factorization needs to be run in multi-times with different initial solutions.

In recent years, more formats of NMF and its variants have been proposed. Besides basic two-factor NMF, three-factor NMF (Ding et al., 2006) is proposed and widely used. The format is $\mathbf{X}\approx\mathbf{USV}$. Factored matrix $\mathbf{V}$ can not only absorb scale difference between $\mathbf{U}$ and $\mathbf{S}$, but also indicate the relationship between identified modules. Meanwhile, many constraints have been also applied to NMF framework. Ding et al. (Ding et al., 2006) imposed orthogonal constraints $\text{‖}\mathbf{X}-\mathbf{UV}\text{‖}^{2},\text{ }s.t.\text{ }\mathbf{VV}^{T}=\mathbf{I}$, and orthogonal NMF leads to rigorous clustering interpretation. Mohammadiha et al. (Mohammadiha and Leijon, 2009) imposed sparseness constraints $\text{‖}\mathbf{X}-\mathbf{UV}\text{‖}^{2}+\beta\text{‖}\mathbf{V}\text{‖}_{L_{0}/L_{1}/F}$. Cai et al. (Cai et al., 2011) imposed graph-regularized constraints $\text{‖}\mathbf{X}-\mathbf{UV}\text{‖}^{2}+\lambda\mathrm{tr}\left(\mathbf{VLV}^{T}\right)$ , it can incorporates prior knowledge such as interaction networks between features or correlation of samples in data $\mathbf{X}$. Zhang et al. (Zhang et al., 2012) proposed a joint NMF (jNMF) method to decomposes *n* data matrices. Each matrix $\mathbf{X}_{i}$, $i=1,2,...,n$ can be decomposed into a common matrix $\mathbf{U}$ and individual matrix $\mathbf{V}_{i}$. So jNMF can simultaneously integrate several genomic data across the same set of samples$\sum_{i} \left\|\mathbf{X}_{i}-\mathbf{U}_{i}\mathbf{V}\right\|^{2}$.

## Supplementary Note 2. Python Package of CBP-JMF

Here is a detailed description of parameters when using CBP-JMF:

1. The python package we established for CBP-JMF can be run as follows: establish CBP_JMF_Solver in CBP-JMF package and multi-run matrix factorization.

CBP_JMF_obj=CBP_JMF.CBP_JMF_Solver(X_input=X_input,rank=4,n_iter=50,beta=10,omega=100000,corr_matrix=corr_test,labeled_count=labeled_count,subgroup=subtype,multi_runtimes=10,consensus_runtimes=10,labeled_sample_list=labeled_sample_list,unlabeled_sample_list=unlabeled_sample_list,z_threshold=2.0,col_color=col_color)

There are several parameters to pass into it.

1. *Rank*: number of latent component of input matrix. Rank k is usually decided in advance, if users don’t have appropriate rank or users want to know the best rank, CBP-JMF provides a consensus clustering for users to determine k. In our data, samples are classified into four subtypes, so rank is set as 4.
2. $\beta,\omega$: represent the importance of graph laplacian regularization part and weight part respectively. Both two parameters are decided according to the relative size of norm of input data matrices and norm of graph laplacian. CBP-JMF provides users a *parameter_recommendation* function to obtain appropriate $\beta,\omega$. In our data, $\beta,\omega$ are set as 10, 100000 respectively.
3. *n_iter*, *multi_runtimes*: *n_iter* represents the iterations of optimization in a group of initial solution matrices $\mathbf{U}$, $\mathbf{V}$, $\mathbf{S}$. *multi_runtimes* represents the times of randomly initializing solution matrices. Larger *n_iter* and *multi_runtimes* may get a better solution**,** but will cost more time. In our data, n_iter and multi_runtimes are set as 50, 10 respectively.
4. *z_threshold*: threshold to get each module's members. For each column of Ui, we calculated z-scores and decided the module members through a threshold cutoff. In our data, *z_threshold* are set as 2.0.
5. Predict unlabeled samples' label and extract module members of each subtype. After step 1, we will get unique$\mathbf{U}^{\left(1\right)},\mathbf{U}^{\left(2\right)},...,\mathbf{U}^{\left(P\right)}$,$\mathbf{S}^{\left(1\right)},\mathbf{S}^{\left(2\right)},...,\mathbf{S}^{\left(P\right)}$ and a common $\mathbf{V}$. In our data, from matrix $\mathbf{U}^{\left(p\right)},p=1,2,...P$, we can obtain each module’s members (genes and miRNAs), from matrix $\mathbf{V}$, we will know each subtype’s corresponding module and classify unlabeled samples into four subtypes.
6. Prediction_result=CBP_JMF_obj.predict_subtype(V_min)
7. CBP_JMF_obj.subtype_module_correspond(V_min)
8. module_from_X1=CBP_JMF_obj.getmodulegene(X1_feature,U_min[0])
9. module_from_X2=CBP_JMF_obj.getmodulegene(X2_feature,U_min[1])

If users are uncertain about parameters rank, $\beta$ and $\omega$, CBP-JMF can help users decide appropriate parameters with the following commands.

1. CBP_JMF_obj.parameter_recommendation()
2. cophenet=CBP_JMF_obj.cophenetic(rank_start=2,rank_end=7)

This function is based on cophenetic coefficient (Brunet et al., 2004) of consensus clustering with different rank and the relative size of norm of input data matrices and norm of graph laplacian. Supplementary Figure 1 shows the selection of rank in example of BRCA by CBP-JMF.

1. The calculation of inter-group samples adjacency matrices $W^{p}$.

We calculate $W^{p}$ using single omics data such as CNV data, miRNA data, et al. We represent the omics data to calculate $W^{p}$ is $O$. In addition, we represent the number of labeled samples and unlabeled samples are *num_labeled* and *num_unlabeled* respectively. Firstly, we calculate the Pearson correlation coefficient (PCC) between any labeled sample pairs with $O$ and denote the correlation matrix as $W$. Secondly, we obtain $W^{a}$ through retaining the PCC values between sample pairs from the same disease subtype and set other values in $W$ to zero. Thirdly, if *num_unlabeled* is not equal to 0, we obtain $ratio=num\_labeled/num\_unlabeled$ and $W^{p}=\alpha\times ratio\times\left(W-W^{a}\right)$. Otherwise, $W^{p}=\alpha\times\left(W-W^{a}\right)$. The parameter $\alpha$ controls the distance between inter-group samples. In our experiment, we set $\alpha\text{=0.1}$.

## Supplementary Note 3. The Relationship Between Subtypes and Modules

In Supplementary Figure 3, we plot the heat map of matrix $\mathbf{V}^{L}$ in one run for BRCA. Among four rows of $\mathbf{V}^{L}$, samples of Luminal A have their highest value in the third row, samples of Luminal B have their highest value in the fourth row, Basal-like’s samples have their highest value in the first row, and HER2-enriched’s samples have their highest value in the second row. Therefore, the corresponding relationship between subtypes and rows of V are: Luminal A <–> row 3, Luminal B <-> row 4, Basal-like <-> row 1, HER2-enriched <-> row 2.

## Supplementary Note 4. Predict Label of Unlabeled Samples

CBP-JMF can be used to assign an unlabeled sample $i$ to a labeled group. For $\mathbf{v}_{i}^{UL}$ is the *i-*th column vector of $\mathbf{V}^{UL}$, CBP-JMF classifies $i$ into the corresponding group $j$ of which latent feature $\mathbf{v}_{ji}^{UL}$ gets the maximum value of $\mathbf{v}_{i}^{UL}$. The clustering result is usually evaluated by comparing the obtained label of each sample with the label provided by the dataset. Here, CBP-JMF integrated three quantification indexes: Purity, Adjusted Rand Index (ARI) and Normalized Mutual Information (NMI) to evaluate the result.

In the case of BRCA, we classify unlabeled samples into four subtypes according to the value of $\mathbf{V}$ matrix, we use Supplement Figure 3 as an example, if an unlabeled sample has its highest value in the first row of $\mathbf{V}^{UL}$ (unlabeled part of $\mathbf{V}$), then it will be classified to Basal-like subtype.

After classifying unlabeled samples into 4 subtypes, we did survival analysis on unlabeled samples. For comparison, we also performed survival analysis using only gene expression data as input (Equation (1)) and without supervised graph laplacian constraints (Equation (2)) in Figure 4.

$\begin{matrix} \min\left\|\mathbf{X}^{\left(1\right)}-\mathbf{U}^{\left(1\right)}\mathbf{S}^{\left(1\right)}\mathbf{V}\right\|_{F}^{2}+\beta\left\{tr\left[\mathbf{V}^{L}\mathbf{L}^{a}\left(\mathbf{V}^{L}\right)^{T}\right]-tr\left[\mathbf{V}^{L}\mathbf{L}^{p}\left(\mathbf{V}^{L}\right)^{T}\right]\right\}+\omega\left\|\mathbf{\Pi}\right\|^{2} \\ s.t. \forall p,\mathbf{U}_{ij}^{\left(p\right)}\geq0,\mathbf{V}_{ij}\geq0 \end{matrix}$ (1)

$\begin{matrix} \min_{\left\{\mathbf{U}^{(p)}\right\}_{p=1}^{P},\left\{\mathbf{S}^{(p)}\right\}_{p=1}^{P},\mathbf{V}} \sum_{p=1}^{P} \mathbf{\pi}^{\left(p\right)}\left\|\mathbf{X}^{\left(p\right)}-\mathbf{U}^{\left(p\right)}\mathbf{S}^{\left(p\right)}\mathbf{V}\right\|_{F}^{2}+\omega\left\|\mathbf{\Pi}\right\|^{2} \\ s.t. \forall p,\mathbf{U}_{ij}^{\left(p\right)}\geq0,\mathbf{V}_{ij}\geq0,\mathbf{\pi}^{\left(p\right)}\geq0,\sum_{p=1}^{P} \mathbf{\pi}^{\left(p\right)}=1 \end{matrix}$ (2)

In our example of using BRCA data, after 50 times randomly initializing solution and optimizing, CBP-JMF achieved purity: 0.85±0.11, ARI: 0.82±0.13, NMI: 0.80±0.09. The result proves that CBP-JMF has great clustering performance.

# Supplementary Figures and Tables


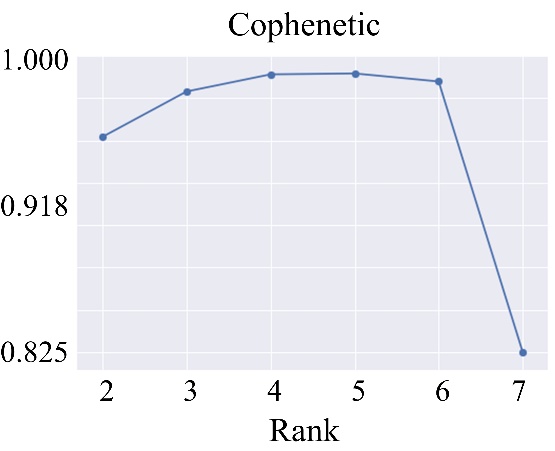


Supplementary Figure 1. Cophenetic coefficient in our example data at different rank. The higher coefficient, the better performance consensus clustering achieves. Rank at 4 or 5 is the best rank for our example data and this proves the correctness of rank=4 in our experiment.


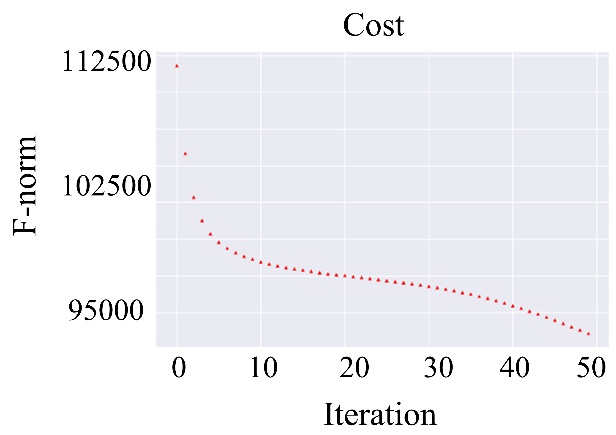


**Supplementary Figure 2.** Objective function of CBP-JMF during optimization.


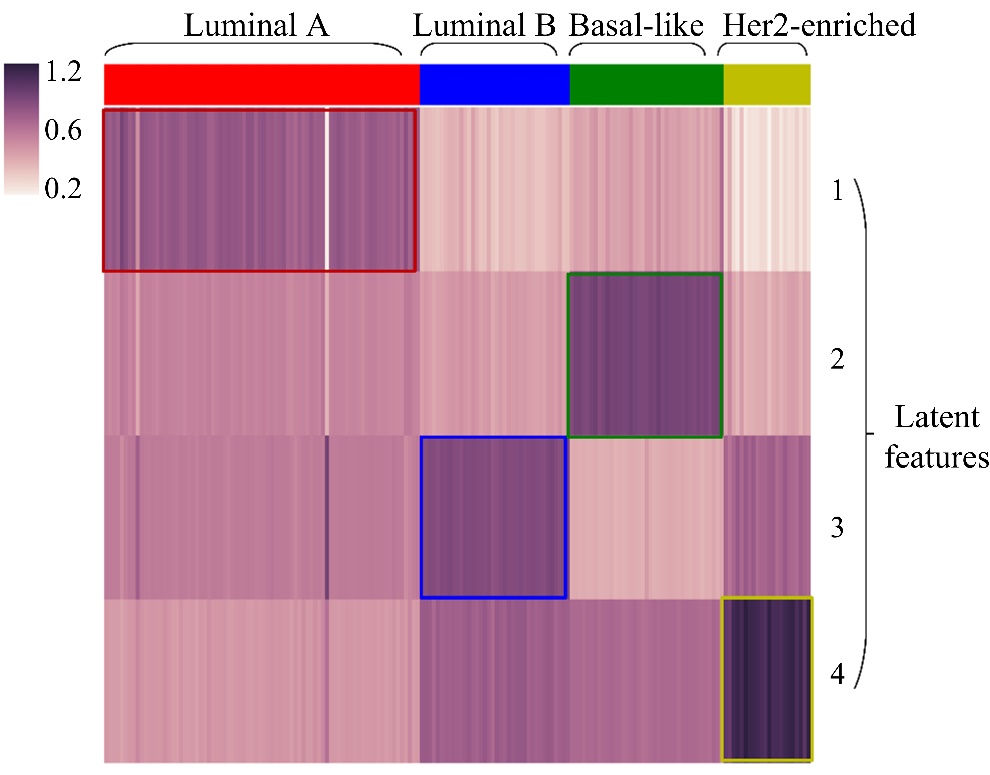


**Supplementary Figure 3.** Heatmap of $\mathbf{V}^{L}$.


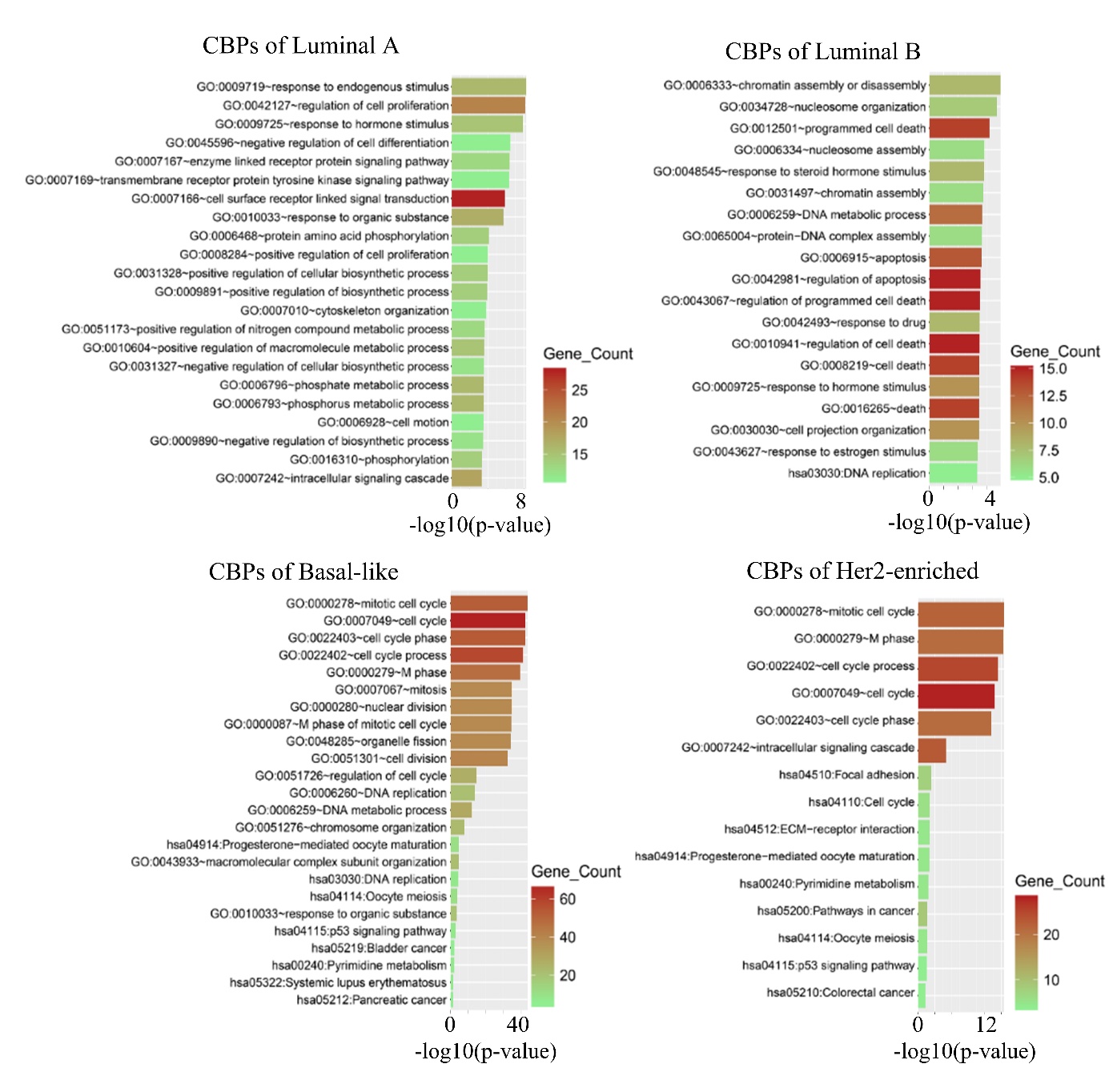


Supplementary Figure 4. Functional enrichment for CBPs of four different subtypes of BRCA.


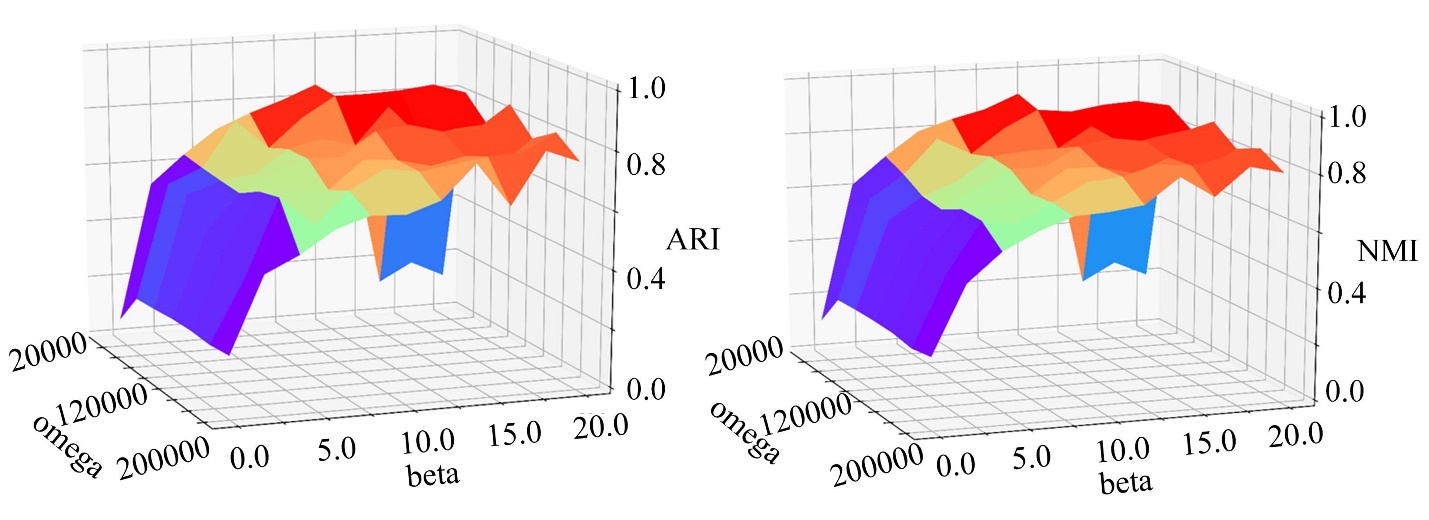


Supplementary Figure 5. The impact of parameter $\beta$ and $\omega$ on quantification indexes ARI and NMI when assigning labels to unlabeled samples.

# References

Brunet, J. P., Tamayo, P., Golub, T. R., and Mesirov, J. P. (2004). Metagenes and molecular pattern discovery using matrix factorization. *Proc. Natl. Acad. Sci. U. S. A.* 101, 4164–4169. doi:10.1073/pnas.0308531101.

Cai, D., He, X., Han, J., and Huang, T. S. (2011). Graph Regularized Nonnegative Matrix Factorization for Data Representation. *IEEE Trans. Pattern Anal. Mach. Intell.* 22, 1548–1560. doi:10.1007/s10489-019-01539-9.

Ding, C., Li, T., Peng, W., and Park, H. (2006). Orthogonal nonnegative matrix tri-factorizations for clustering. *Proc. ACM SIGKDD Int. Conf. Knowl. Discov. Data Min.* 2006, 126–135. doi:10.1145/1150402.1150420.

Lee, D. D., and Seung, H. S. (1999). Learning the parts of objects by non-negative matrix factorization. *Nature* 401, 788–791. doi:10.1038/44565.

Mohammadiha, N., and Leijon, A. (2009). Nonnegative matrix factorization using projected gradient algorithms with sparseness constraints. *IEEE Int. Symp. Signal Process. Inf. Technol. ISSPIT 2009*, 418–423. doi:10.1109/ISSPIT.2009.5407557.

Wang, Y.-X., and Zhang, Y.-J. (2013). Nonnegative matrix factorization: A comprehensive review. *IEEE Trans. Knowl. Data Eng.* 25, 1336–1353. doi:10.1109/TKDE.2012.51.

Zhang, S., Liu, C. C., Li, W., Shen, H., Laird, P. W., and Zhou, X. J. (2012). Discovery of multi-dimensional modules by integrative analysis of cancer genomic data. *Nucleic Acids Res.* 40, 9379–9391. doi:10.1093/nar/gks725.

Brunet, J. P., Tamayo, P., Golub, T. R., and Mesirov, J. P. (2004). Metagenes and molecular pattern discovery using matrix factorization. *Proc. Natl. Acad. Sci. U. S. A.* 101, 4164–4169. doi:10.1073/pnas.0308531101.

Cai, D., He, X., Han, J., and Huang, T. S. (2011). Graph Regularized Nonnegative Matrix Factorization for Data Representation. *IEEE Trans. Pattern Anal. Mach. Intell.* 22, 1548–1560. doi:10.1007/s10489-019-01539-9.

Ding, C., Li, T., Peng, W., and Park, H. (2006). Orthogonal nonnegative matrix tri-factorizations for clustering. *Proc. ACM SIGKDD Int. Conf. Knowl. Discov. Data Min.* 2006, 126–135. doi:10.1145/1150402.1150420.

Lee, D. D., and Seung, H. S. (1999). Learning the parts of objects by non-negative matrix factorization. *Nature* 401, 788–791. doi:10.1038/44565.

Mohammadiha, N., and Leijon, A. (2009). Nonnegative matrix factorization using projected gradient algorithms with sparseness constraints. *IEEE Int. Symp. Signal Process. Inf. Technol. ISSPIT 2009*, 418–423. doi:10.1109/ISSPIT.2009.5407557.

Wang, Y.-X., and Zhang, Y.-J. (2013). Nonnegative matrix factorization: A comprehensive review. *IEEE Trans. Knowl. Data Eng.* 25, 1336–1353. doi:10.1109/TKDE.2012.51.

Zhang, S., Liu, C. C., Li, W., Shen, H., Laird, P. W., and Zhou, X. J. (2012). Discovery of multi-dimensional modules by integrative analysis of cancer genomic data. *Nucleic Acids Res.* 40, 9379–9391. doi:10.1093/nar/gks725.
